# Field emission: calculations supporting a new methodology of comparing theory with experiment

**DOI:** 10.1098/rsos.220748

**Published:** 2022-11-30

**Authors:** Sergey V. Filippov, Anatoly G. Kolosko, Eugeni O. Popov, Richard G. Forbes

**Affiliations:** ^1^ Division of Plasma Physics, Atomic Physics and Astrophysics, Ioffe Institute, St. Petersburg 194021, Russia; ^2^ Advanced Technology Institute & School of Computer Science and Electronic Engineering, University of Surrey, Guildford, Surrey GU2 7XH, UK

**Keywords:** field electron emission science, extended Murphy–Good FE theory, field emission theory and experiment, notional cap-area efficiency, field emission decision table, AHFP equation pre-exponential parameter

## Abstract

This paper provides a demonstration-of-concept of a new methodology for comparing field electron emission (FE) theory and experiment. It uses the parameter *κ* in the mathematical equation *I*_m_ = *CV*_m_*^κ^* exp[–*B*/*V*_m_] (where *B* and *C* are weakly varying or constants) that is taken to describe how measured current *I*_m_ depends on measured voltage *V*_m_ for electronically ideal FE systems (i.e. systems that (i) have constant configuration during voltage application and (ii) have *I*_m_(*V*_m_) given by the emission physics alone). Experimental parameter values (*κ*_m_) are used to compare two alternative FE theories, for which allowable (but different) *κ* ranges have been established. At present, contributions to the ‘total theoretical *κ*’ made by voltage dependence of notional emission area are not well known: simulations reported here provide data about four commonly investigated emitter shapes. The methodology is then applied to compare 1928/1929 Fowler–Nordheim (FN) FE theory and 1956 Murphy–Good (MG) FE theory. It is theoretically certain that the 1956 theory is ‘better physics’ than the 1928/1929 theory. As in previous attempts to reach known correct theoretical conclusions by experimentally based argument, the new methodology tends to favour MG FE theory, but is formally indecisive at this stage. Further progress needs better methods of establishing error limits and of measuring *κ*_m_.

## 1. Introduction

The work reported here contributes to a much wider project, recently outlined elsewhere [1], that aims to put field electron emission (FE) theory on to a more satisfactory scientific basis. As part of this wider project, it is wished to (i) improve existing methodologies for analysing FE current–voltage data and (ii) improve existing procedures for using analysis results to derive useful scientific conclusions.

Sections 2–4 set out some detailed background to the present work; the remaining sections then present technical details and conclusions.

## 2. General background

### 2.1. Issues related to overall field emission system behaviour

The term *FE system* is defined to include all aspects of the experimental system that can affect the relationship between *measured current I*_m_ and *measured voltage V*_m_, including: emitter ‘configuration’ (composition, geometry and surface condition); the mechanical, geometrical and electrical arrangements in the vacuum system; all aspects of the electronic circuitry and of all electronic measurement instruments; the emission physics; and ALL relevant physical processes that might be taking place (for example, the generation of field-emitted vacuum space charge, Maxwell stress-induced reversible changes in emitter geometry and adsorbate atom dynamics).

This *I*_m_(*V*_m_) relationship can be discussed as follows. It is assumed that there exists a definite (albeit unknown) relationship *I*_m_(*F*_C_) between *I*_m_ and the magnitude *F*_C_ of the electrostatic field at some defined *characteristic location* ‘C’ on the emitter surface, and that the relationship between *F*_C_ and *V*_m_ can be written

2.1 $$F_{C}=\frac{V_{m}}{\zeta_{C}},$$

where *ζ*_C_ is a parameter called the (characteristic) *local (measured) voltage conversion length (LVCL)*. The LVCL *ζ*_C_ (discussed further below) is a system characterization parameter. The description ‘local’ indicates that it applies to some specific location on the emitter surface and involves the local field magnitude at that location. An LVCL has the dimensions of length, but does not in practice correspond to any geometrical length in the FE system.

An FE system is termed *electronically ideal* if (i) there are no changes in system configuration as a result of application of voltage and (ii) there are no voltage- or current-dependent changes in the value of any LVCL as a result of ‘system complications'. The most commonly discussed system complications are series resistance, field-emitted vacuum space charge and system geometry change due to Maxwell stresses, but there are many others. For a list of the 12 types of system complication currently known, see https://doi.org/10.13140/RG.2.2.10294.57927, and for a discussion of some of the resulting difficulties, see [2].

Paradigm examples of electronically ideal systems are the 1920s and 1930s systems where the emitter and all aspects of the emitter-mounting arrangements are metal, and the system is operated under conditions of good high vacuum and at fields where there is no significant field-emitted vacuum space charge. Many modern systems are electronically ideal, over all or part of their voltage working range, but many others are not.

Normally, some form of *validity check* needs to be applied to establish whether experimental data are likely to have been derived from an electronically ideal system and hence can be validly interpreted using standard data-analysis techniques such as Fower–Nordheim (FN) plots. There currently exist three forms of validity check, namely: (i) apparent linearity of an FN or similar data-analysis plot; (ii) the Orthodoxy Test [3] and (iii) the so-called ‘magic emitter test’ (see ResearchGate presentation just cited). With electronically non-ideal systems, the interpretation of measured FE current–voltage characteristics can be extremely complex and often impossible to carry out exactly in the present state of research knowledge.

For electronically ideal FE systems, the interpretation problem becomes part of FE emission physics, with the value of the characteristic LVCL determined *in principle* by the zero-current electrostatics of the system geometry (but in practice nearly always by experiment).

We make the point that this paper is NOT directly about predicting the outcome of FE experiments of the type usually carried out. Rather, it is aimed at the question of how to design experiments (and experimental apparatus) so that useful and reliable comparisons can be made between theory and experiment. Further, it describes the start of a research journey, not the conclusion of one.

Obviously, normal scientific thinking about journeys of this kind is that one needs to avoid using experimental systems and conditions that are more complicated than necessary. In particular, one should in principle avoid experiments on emitters, such as so-called large-area field electron emitters (LAFEs), that have very many individual emission sites, with a statistical distribution of characterization parameters.

Hence, also, in a journey of this kind in FE contexts, it is desirable to only use experimental data from electronically ideal systems. Although the LVCL definition above applies to all systems, it will in this paper only be applied to electronically ideal systems (for which it can be assumed that *ζ*_C_ is constant).

We also note that the parameter *ζ*_C_ (which is best measured in nm) is the reciprocal of the parameter *β* apparently first introduced by Dyke and Trolan in 1953 [4] and best measured in m^–1^.

We prefer using LVCLs rather than values of the Dyke–Trolan *β*, partly in order to avoid potential confusion resulting from the widespread use (in FE literature) of the symbol ‘*β*’ to denote dimensionless field enhancement factors, partly because we think a parameter measured in nm is easier to work with than one measured in m^–1^. In particular, if equation (2.1) is inverted into the alternative form

2.2 $$V_{m,\mathrm{on}}=\zeta_{C}F_{C,\mathrm{on}},$$

then *ζ*_C_ has a simple physical interpretation as the factor that links the onset measured voltage *V*_m,on_ to the onset value *F*_C,on_ of the characteristic local field magnitude. For example, for a 4.50 eV emitter, *F*_C,on_ is around 2 V nm^−1^, so if *ζ*_C_ had the value 1000 nm, then the onset measured voltage would be predicted as around 2000 V.

### 2.2. Issues related to emission physics and data analysis

For the last 90 years, since the publication in 1928 [5] of the ground-breaking (but seriously flawed [6,7]) paper of FN, most FE theory has been developed in the context of so-called *smooth planar metal-like emitter (SPME) methodology*. This methodology disregards the existence of atomic structure (including the possible role of surface atom orbitals in FE theory), uses Sommerfeld-type free-electron theory and treats emitters as having smooth, structureless planar surfaces of large lateral extent. However, it is possible to treat needle-shaped emitters that are ‘not too sharply pointed’ by using the *planar emission approximation* [1] and carrying out an integration of local emission current density (LECD) over the emitter surface.

As is well known, the main theoretical LECD equations in SPME methodology have been: (i) the original 1928 FN FE equation (as corrected in 1929 [8]), which used an exactly triangular (ET) transmission barrier; and (ii) the 1956 Murphy–Good (MG) FE equation [9], which is based on the Schottky-Nordheim (SN) (or ‘planar image rounded’) transmission barrier.

The most common method of analysing *I*_m_(*V*_m_) data has been the well-known FN plot, introduced by Stern *et al*. in 1929 [8] because these authors thought that an FN plot would be exactly straight. The 1956 MG FE theory is better physics than the 1928/1929 FN FE theory (see [7] for a discussion) and predicts that an FN plot will be slightly curved. This curvature makes it difficult to reach accurate quantitative conclusions from the vertical-axis intercept of the straight line fitted to the experimental data plot (see [10] and Appendix A in [11]). However, a different plot form, the so-called MG plot (see [11] for details) should (in SPME methodology) be ‘very nearly straight’.

However, even for electronically ideal FE systems, it is not expected that an experimental MG plot should be exactly straight. This is because other factors, neglected in MG FE theory (and more generally in SPME methodology), are known to contribute additional voltage dependence and thus to cause departure from strict MG plot linearity. The main factors of this type are (i) the long-known fact [12] that voltage dependence in notional emission area (see below) will be introduced when an experimental or theoretical integration of LECD over the needle surface is carried out and (ii) the need to take surface atom atomic orbitals into account in a complete FE theory.

There are significant fundamental physical difficulties involved in *accurately* incorporating atomic structure into FE theory and this has persuaded the authors to take a strategically different approach to making comparisons between FE theory and experiment. Rather than formulating some specific advanced FE theory and then attempting to test this against experiment, there has been a proposal [13,14] that attempts should be made to fit experimental data for FE systems proven to be electronically ideal to the *mathematical form*

2.3 $$I_{m}=CV_{m}^{\kappa }\exp\left[-\frac{B}{V_{m}}\right],$$

where *B*, *C* and *κ* (also written ‘*k’* in some past work) are parameters that in the first instance have been treated as constants. Two methods of carrying out this fitting have been discussed in [14].

Our present expectation is that *C* and *κ* will be weakly varying functions of field, and that *B* will be a constant or a very weakly varying function of field. Thus, depending on the data-analysis method used, the extracted values of *C* and *κ* may be average values for the range of voltages (and hence the range of characteristic field values) used.

We emphasize that equation (2.3) is a mathematical form used for fitting, *not* a theory of FE. In particular, it should *not* be assumed that exp[–*B*/*V*_m_] is an expression for the Gamow factor (or ‘barrier strength’) that is used in FE theory. We also emphasize again that equation (2.3) is a mathematical form that is being applied in this paper to experimental data from FE systems assumed to be *electronically ideal* (i.e. systems for which the LVCL is constant), *not* to FE *I*_m_(*V*_m_) data in general––for which the LVCL may be a function of measured voltage.

Equation (2.3) has previously been called the ‘empirical FE equation’, but this name now seems potentially misleading. The main previous users of this mathematical form were Abbott & Henderson in 1939 [12] (who used only integral values of *κ*), Forbes in 2008 [13], and Forbes *et al.* in 2019 [14]. Both [13] and [14] allowed *κ* to be non-integral. Thus, this paper employs the first letters of the family names of the main past users and refers to equation (2.3), with non-integral values allowed, as the *AHFP mathematical form* (for measured voltage).

The parameter ‘*κ*’ has been called the ‘pre-exponential voltage exponent’ [14]. However, for an electronically ideal FE system, both the characteristic field *F*_C_ and the related characteristic scaled-field *f*_C_ (defined below) are directly proportional to *V*_m_. Thus, when AHFP-type equations are formulated in terms of these alternative variables, factors of *F*_C_*^κ^* or *f*_C_*^κ^* will appear in the equation. Thus, particularly when discussing electronically ideal FE systems, it is now considered preferable to formally call *κ* the *AHFP equation pre-exponential parameter.* However, for simplicity here, we will sometimes refer to it as the ‘total *κ-value*’.

In principle, our long-term aim *in the context of emission theory* is as follows. After confirming that an FE system under investigation is electronically ideal, and then extracting experimentally derived values of *B*, *C* and *κ*, our aim is to use these values to deduce more detailed information about the nature of FE theory. It is expected that the values of *C* and *κ* will be more useful than the value of *B*, but that trying to interpret extracted values of *C* and *κ* simultaneously would be a difficult endeavour. Current efforts have therefore concentrated on issues related to the extraction and interpretation of values of *κ* alone.

It has been shown by Forbes *et al*. [14], by simulations, that the best average value of *κ* found (for an electronically ideal system) from a plot of ln**{***I*_m_*/V*_m_*Κ*} versus 1/*V*_m_ (a so-called *power-κ plot*) is a sensitive function of both (i) the details of emission theory and (ii) the shape of the field emitter. Hence, if *I*_m_(*V*_m_) measurements of high quality were available for *electronically ideal* FE systems, and the effects of emitter shape and surface condition could be disentangled from these, then a measured value of *κ* should provide a method of comparing different FE theories with experiment. Here we report some steps towards at least assessing emitter-shape effects.

An experimental alternative would be to make reliable direct measurements of LECD, but this seems to be significantly more difficult experimentally.

As part of the overall process, we need to know: (i) what physical effects contribute to the experimental value (*κ*_m_) of *κ*; (ii) what is the size of each contribution (or what are the limits within which each contribution size is likely to lie); and (iii) what information is already available in the literature. Existing knowledge about these things is reviewed in §3, where it will become apparent that a gap in our knowledge is good understanding of how the voltage dependence of the notional emission area depends on the emitter shape and on an assumed work-function value. An aim of this paper is to report the result of simulations relating to this knowledge gap. This is done in §§3 and 4. However, we need first to provide, in §3.1, some additional details of existing theory.

Finally, here, we need to point out that equation (2.3) is not the only mathematical form to have been suggested for fitting to experimental results. On the basis of a theoretical discussion based on so-called fractional-dimensional space, Zubair *et al*. [15] have suggested the mathematical form

2.4 $$I=CV^{2a}\exp\left[-\frac{B}{V^{a}}\right],$$

where *V* and *I* are a voltage and a current that appear in their theory, and *a* is a parameter that they call the *fractional space dimensionality parameter*.

The present authors have significant hesitations about the relevance of the theoretical methodology used to derive this formula. It would be outside the scope of this paper to discuss details here, but we plan to raise them privately with Zubair *et al.* at a later time. However, whatever the merits of its derivation, it is clear that equation (2.4) can be regarded as an alternative mathematical form to which experimental data might be fitted.

Zubair *et al*. argue that this is an appropriate mathematical form for experimental data taken from rough surfaces. Equation (2.4) would also, in principle, be an alternative form for fitting data from electronically ideal ‘flat-smooth-surface’ FE systems of the kind that we are interested in. In this context, we make two points. First, it is possible to envisage that we shall have to explore (in sequence) a number of mathematical forms of gradually increasing complexity. Form (2.4) is more complex than form (2.3), but it is possible to argue that a better choice of ‘second preferred form’ might be

2.5 $$I_{m}=CV_{m}^{a}\exp\left[-\frac{B}{V_{m}^{b}}\right],$$

where *a* and *b* are arbitrary mathematical parameters. Second, the best approach seems to be to thoroughly explore the usefulness of equation (2.2) before moving on to anything more complicated.

## 3. Emission theory background

### 3.1. Summary of extended Murphy–Good field electron emission current–voltage theory

For our simulations, we use the *extended Murphy–Good (EMG)* formulation (see [11]) of FE theory. The local *kernel current density for the SN barrier* (*J*_kL_^SN^) is given by

3.1 $$J_{\mathrm{kL}}^{\mathrm{SN}}=a\phi^{-1}F_{L}^{2}\exp\left[-\frac{v_{F}b\phi^{3/2}}{F_{L}}\right],$$

where the symbols have their usual meanings [16]. This equation can be put into *scaled format* by defining three parameters:

3.2 $$\eta (f)\equiv bc_{S}^{2}\phi^{\text{–}1/2},$$

3.3 $$\theta (f)\equiv ac_{S}^{\text{–}4}\phi^{3},$$

3.4 $$f_{L}\equiv c_{S}^{2}\phi^{\text{–}2}F_{L},$$

where *c*_S_ [≡ (*e*^3^/4π*ε*_0_)^1/2^)] is the *Schottky constant* and *f*_L_ is the local *scaled-field* for a barrier of zero-field height *ϕ*. Using these yields the *scaled format expression*:

3.5 $$J_{\mathrm{kL}}^{\mathrm{SN}}=\theta f_{L}^{2}\exp\left[-\frac{v(f_{L})\cdot \eta }{\;f_{L}}\right].$$

In EMG theory, the *LECD J*_L_^EMG^ is written

3.6 $$J_{L}^{\mathrm{EMG}}=\lambda_{L}^{\mathrm{SN}}J_{\mathrm{kL}}^{\mathrm{SN}},$$

where the parameter *λ*_L_^SN^ is a *prediction uncertainty factor* of unknown value and functional form. It formally takes account of all relevant physics not included in 1956 MG FE theory, including atomic-level wave function effects and band structure effects, and also incorporates the usual MG pre-exponential correction factor t_F_ ^–2^ and the MG temperature correction factor.

If the kernel current density *J*_kL_^SN^ is integrated over the surface of a needle-shaped emitter, using the planar emission approximation, then the resulting calculated *notional emission current I*_n_^SN^ can be written in the form

3.7 $$I_{n}^{\mathrm{SN}}=A_{\mathrm{nC}}^{\mathrm{SN}}J_{\mathrm{kC}}^{\mathrm{SN}},$$

where *J*_kC_^SN^ is the value of the kernel current density at some surface location ‘C’ that characterizes the emitter. (In modelling, ‘C’ is usually taken as the emitter apex). The *notional emission area A*_nC_^SN^ is defined by equation (3.7); it depends on the chosen emitter shape and work-function distribution, on the assumed nature of the barrier (here, the SN barrier), and on the choice of location ‘C’.

We may suppose that, for the chosen emitter model, there would be a true (but unknown) model emission current *I*_tm_, and that we can write:

3.8 $$I_{\mathrm{tm}}=\lambda_{J}^{\mathrm{SN}}I_{n}^{\mathrm{SN}},$$

where *λ_J_*^SN^ is a *prediction uncertainty factor for current density*, of the same general kind as *λ*_L_^SN^. The factor *λ_J_*^SN^ will depend on many things, but we specifically show the barrier label. This is because the interpretation of experimental results (in particular, FN and related plots) requires an assumption about the nature of the transmission barrier.

In reality, when attempting to accurately model the ‘predicted’ emission current *I*_p_ from a real emitter, it is inevitable (at least at present) that the model emitter will not be an accurate model for the real emitter. Hence, a second source of prediction uncertainty is introduced, and we can write

3.9 $$I_{p}=\lambda_{\mathrm{EM}}^{\mathrm{SN}}I_{\mathrm{tm}}=\lambda_{\mathrm{EM}}^{\mathrm{SN}}\lambda_{J}^{\mathrm{SN}}I_{n}^{\mathrm{SN}},$$

where *λ*_EM_^SN^ is a prediction uncertainty factor related to emitter-model inadequacy.

Using equation (3.7) yields

3.10 $$I_{p}=\lambda_{\mathrm{EM}}^{\mathrm{SN}}\lambda_{J}^{\mathrm{SN}}I_{n}^{\mathrm{SN}}=\lambda_{\mathrm{EM}}^{\mathrm{SN}}\lambda_{J}^{\mathrm{SN}}A_{\mathrm{nC}}^{\mathrm{SN}}J_{\mathrm{kC}}^{\mathrm{SN}}.$$

This can be simplified by defining a new area-like parameter *A*_fC_^SN^, *the formal emission area for the SN barrier* (as defined by location ‘C’), by

3.11 $$A_{\mathrm{fC}}^{\mathrm{SN}}=\lambda_{\mathrm{EC}}^{\mathrm{SN}}\lambda_{J}^{\mathrm{SN}}A_{\mathrm{nC}}^{\mathrm{SN}}.$$

Equation (3.10) can then be rewritten as

3.12 $$I_{p}=A_{\mathrm{fC}}^{\mathrm{SN}}J_{\mathrm{kC}}^{\mathrm{SN}}.$$

Leakage current, if present, can be modelled by an additional correction factor included within *λ*_EM_^SN^.

We now assert that (for the electronically ideal systems under discussion) the ‘formally predicted’ emission current *I*_p_ can be identified with the measured current *I*_m_, and hence (using equation (3.5)) we can write

3.13 $$I_{m}=A_{\mathrm{fC}}^{\mathrm{SN}}J_{\mathrm{kC}}^{\mathrm{SN}}=A_{\mathrm{fC}}^{\mathrm{SN}}\theta f_{C}^{2}\exp\left[-\frac{v(f_{C})\cdot \eta }{\;f_{C}}\right],$$

where *f*_C_ is the scaled-field at location ‘C’.

In equation (3.13), *I*_m_ is well defined and *J*_kC_^SN^ is well defined in principle (if location ‘C’ is known, and the values of *ϕ* and *f*_C_ are stated). Hence, the formal area *A*_fC_^SN^ is well defined in principle. It is this *formal* emission area that is extracted from experiments, typically by using FN plots.

For an electronically ideal FE system, the parameter *f*_C_ can also be written *f*_C_ = *V*_m_/*V*_mR,_ where *V*_mR_ is the *reference measured voltage* needed to pull the top of the SN barrier down to the Fermi level, at location ‘C’.

Our assumption is that, in a first approximation (at least over defined ranges of measured voltage *V*_m_), $A_{\mathrm{nC}}^{\mathrm{SN}}$ can be written in the form

3.14 $$A_{\mathrm{nC}}^{\mathrm{SN}}=C_{A}V_{m}^{\kappa A}=\{C_{A}V_{\mathrm{mR}}^{\kappa A}\}f_{C}^{\kappa A},$$

where *C_A_* and *κ_A_* are parameters associated with the shape and work-function characteristics of the emitter. As already indicated, we expect these parameters either to be constants, or (more likely) to be weakly varying functions of apex field and hence of measured voltage.

The discussion above is given specifically for the SN barrier and EMG theory, but generalized versions of the theory can be given for other barrier forms.

### 3.2. Individual contributions

For clarity, in this section, the individual contributions to the total *κ*-value are denoted by the symbol Δ*κ*. The factors currently known to affect the experimental *κ-*value are as follows.

*A. Emitter band structure. A three-dimensional free-electron model of emitter band structure, as used in deriving the 1928/1929 FN and 1956 MG FE equations, makes a contribution Δ*κ* = 2. Models involving lower dimensionality will make, and situations involving quantum confinement may make, a contribution less than 2. It is not clearly known what the effects of more complicated real band structures, such as the ‘many-valley’ band structure of silicon [17], are expected to be.

*B. Barrier form (i.e. shape). As has been shown in [13,14,18,19], there exist formulations of FE theory in which an SN barrier makes a contribution to *κ*. (For an emitter with local work function equal to 4.50 eV, Δ*κ* ≈ –0.773.) Barriers similar in form to the SN barrier may also behave in this way.

*C. Atomic-level structure. In Oppenheimer's approach [20,21], FE is interpreted as the field ionization (FI) of the atomic-level orbitals of surface metal atoms. The existing standard theory of the FI of a hydrogen atom [21] is equivalent to setting Δ*κ* = –1. It might reasonably be assumed that an FE theory that in effect involved FI out of surface metal atoms might make a contribution somewhere in the range –1 ≤ Δ*κ* < 0. Some relevant work (e.g. [20–24]) currently exists, but greater depth is probably needed.

*D. Voltage dependence in the notional emission area. As discussed above, a formula for *notional* emission current *I*_n_ will involve a notional emission area *A*_n_, and this will usually have field and voltage dependence. Details will depend on the chosen emitter shape and the work-function distribution. There is some relevant work, e.g. [16,18,19,25,26], but no good general knowledge about the range of Δ*κ*-values involved. The present work aims to help fill this gap.

*E. Other physical factors included in the prediction uncertainty factor (λ_J_) related to emission theory. As indicated above, correction factors related to other physical effects (for example, a temperature correction factor) are formally included in *λ_J_*. These will or may contribute to *κ*. Our present understanding is that––certainly for bulk metals with conventional three-dimensional band structures––contributions due to these ‘other factors’ are likely to be relatively small and hence can be disregarded for the time being. It is currently not clear whether this is also likely for materials with non-conventional band structures.

*F. The prediction uncertainty factor (λ_EM_*) related to emitter-model inadequacy.* When predicting emission currents, if an inadequate emitter model is used, then it is likely that an incorrect total *κ*-value will be predicted and that a correction will be necessary. At present, there is no useful classified knowledge about *λ*_EM_ or this correction, and this paper does not discuss this issue in detail.

Table 1 provides a summary of existing knowledge relating to the AHFP pre-exponential exponent *κ.* Experimental data has been included only if the related FE experimental system is known or thought to be electronically ideal.

**Table 1.** Research results relating to the pre-exponential parameter *κ* in the AHFP mathematical equation, for electronically ideal FE systems. For theoretical sources, the labels ‘A’ (etc.) in the ‘category’ column indicate which of the individual contributions noted in the text were taken into account.

row	year	ref.	structure	category	total *κ*-value
1	1928	Oppenheimer [20,21]	surface metal atom	Theor (C)	0.25
2	1928	Millikan & Lauritsen [27], Lauritsen [28]	thoriated tungsten cylinder	Expt	0
3	1928	Fowler & Nordheim [5]	SPME^a^	Theor (A)	2.00
4	1929	Stern *et al*. [8] (based on re-plot of Fig. 2 in [29])	thoriated tungsten cylinder	Expt	2
5	1929	Stern *et al*. [8] (based on re-plot of results in [30])	pointed tungsten wire	Expt	2
6	1939	Abbott & Henderson [12]	pointed tungsten wire^b^	Expt	4
7	1939	Abbott & Henderson [12]	SPME + PEA^a^	Theor (A + D)	3
8	1958	Landau & Lifschitz [22]	hydrogen atom	Theor (C)	–1.00
9	2008	Forbes [13]	SPME (*ϕ* = 4.50 eV)	Theor (A + B)	1.23
10	2014	Jensen [18,19]	SPME + PEA, HSP	Theor (A + B + D)	1.99 to 2.13
11	2019	Popov *et al*. [31]	CNT LAFE^c^	Expt	1.65
12	2020	Ang *et al*. [32]^d^	two-dimensional-Dirac/Weyl semimetals	Theor (A + E)	1.00
13	2021	Lepetit [33]	graphene	Theor^e^ ∼(A + B + C + E)	1.53
14	2021	Chan *et al*. [34]	three-dimensional-Dirac/Weyl semimetals	Theor (A + E)	3.00
15	2022	Biswas [35]^f^	SPME + PEA, single tip ‘with generic endform’	Theor (A + B + D)	3–*η*/6 [≍2.23]^g^

^a^SPME = smooth planar metal-like emitter; PEA = planar emission approximation.

^b^In their ‘precise measurements', Abbott & Henderson used ‘point-plane’ geometry.

^c^CNT = carbon nanotube; LAFE = large-area field electron emitter. The use of LAFE experiments is in principle undesirable, but we include this result so that there is at least one modern experimental result.

^d^Also see: https://arxiv.org/abs/2003.14004.

^e^Lepetit's advanced theoretical treatment does not easily fit into the classification system used above.

^f^The argument is also made by Biswas that the behaviour of LAFEs is much more complicated than that of single-tip emitters and involves a contribution to the main exponent.

^g^The value 2.23 applies to an emitting surface with local work function 4.50 eV.

A further account of how notional area depends on apex field has recently been presented by Ramachandran & Biswas [26]. This is discussed in §6.

### 3.3. The concept of notional cap-area efficiency

As already indicated, it is of considerable theoretical interest to know how the notional emission area *A*_nC_ varies as a function of characteristic field *F*_C_, and how this functionality depends on the emitter shape and work-function characteristics. In practice, we use cylindrically symmetric models and take ‘*C*’ as located at the emitter model apex ‘a’. Also, it is more useful to investigate the behaviour of the so-called *notional cap-area efficiency g*_n_ (also called the ‘area factor’) given by

3.15 $$g_{n}=\frac{A_{\mathrm{na}}}{2\pi r_{a}^{2}},$$

where *r*_a_ is the *apex radius of curvature* of the model. Further, it is better to investigate *g*_n_ as a function of the *apex scaled-field f*_a_.

Results will depend on what assumption is made as to the form of the transmission barrier. All discussion here assumes an SN barrier and that the planar emission approximation is being used. (Except that to compile the contributions table below, additional calculations have been carried out, using an ET barrier.)

For the special case of a hemisphere-on-plane (HSP) case, Jensen ([17], see eq. (94), or [18], see eq. (30.19)) has obtained an analytical approximation, which he writes in the form *g*(*F*) ≈ 1/[*b* + 4–*ν*]. In our notation, this becomes

3.16 $$g_{n}(\;f_{a})\approx 1/[\eta /f_{a}+4\text{–}\eta /6],\;\;(\mathrm{HSP}),$$

3.17 $$1/g_{n}\approx (4\text{–}\eta /6)+\eta /f_{a},\;\;(\mathrm{HSP}),$$

where the bracketed acronym indicates the shape model under discussion.

However, what we actually need is the form of the relationship ln{*g*_n_} versus ln{*f*_a_}, so that we can establish the power law dependence of *g*_n_ on *f*_a_ and hence (for electronically ideal systems) on *V*_m_. Straightforward algebraic manipulation leads to the result

3.18 $$\Delta \kappa =\mathrm{dln}\{g_{n}\}/\mathrm{dln}\{\;f_{a}\}\approx 1/[1+\{(4/\eta )\text{–}(1/6)\}f_{a}].$$

For *ϕ* = 4.50 eV, *η* = 4.6368; hence equation (3.18) yields

3.19 $$\Delta \kappa \approx 1/[1+0.696f_{a}],\;\;(\mathrm{HSP}),$$

3.20 $$1/\Delta \kappa \approx 1+0.696f_{a},\;\;(\mathrm{HSP}).$$

So, over the range 0.15 ≤ *f*_a_ ≤ 0.45 (which is the ‘Pass’ range for the Orthodoxy Test [3]), Δ*κ* takes values in the range 0.905 ≥ Δ ≥ 0.761, for the HSP analytical model.

Jensen has also given a formula ([18], see eq. (113)) for the case of a hemi-ellipsoid-on-plane (HEP). He writes his formula as *g*(*F*) ≈ 1/[*b* + 1–*ν*], which in our notation becomes

3.21 $$g_{n}(\;f_{a})\approx 1/[\eta /f_{a}+1\text{–}\eta /6],\;\;\;(\mathrm{HEP}).$$

The formula is stated to hold when the aspect ratio (the ratio of the semi-major to the semi-minor axis, i.e. height divided by base-radius) is large; however, when the aspect ratio approaches unity, then factors that were neglected in the derivation come into play. This means that equation (3.21) as written is not expected to reduce to equation (3.17) when the aspect ratio becomes unity.

Since these are approximate analytical results, we have thought it helpful to check the accuracy of equation (3.17) by means of finite-element electrostatic simulations, as discussed below. We then went on to carry out numerical analyses for emitter shapes of greater practical interest, for which analytical treatment looks either impossible or impracticable.

## 4. Simulation details

In this and the following sections, for historical reasons, the contribution (to the total value of *κ*) made by field/voltage dependence in the notional emission area is denoted by *κ_A,_* rather than by Δ*κ*.

### 4.1. Procedures

To solve the Laplace equation numerically, a commercial finite-element software package (COMSOL v5.3) was used. In addition to the HSP model, we studied three well-known emitter shape models, namely the hemisphere-on-cylindrical-post (HCP), the spherically rounded cone (SRC) and the HEP. Recently [36], it has been shown that the apex field enhancement factors for the hemi-ellipsoidal, paraboloidal and hyperboloidal (with half-angle 5°) posts are similar, so we do not consider paraboloidal and hyperboloidal posts here. Details of our simulation procedure are similar to those found in [14,36].

The emitters (other than the HSP model) are given the following geometric parameters: total height *h* = 4 µm and apex radius of curvature *r*_a_ = 50 nm. This implies that the *apex sharpness ratio σ*_a_ = *h*/*r*_a_ = 80. The SRC emitter has an additional parameter *θ*, which defines the half-angle of the vertex (here, *θ* = 5°). The work function of the emitters was taken as constant across the surface and set to 4.50 eV.

**Figure 1.**
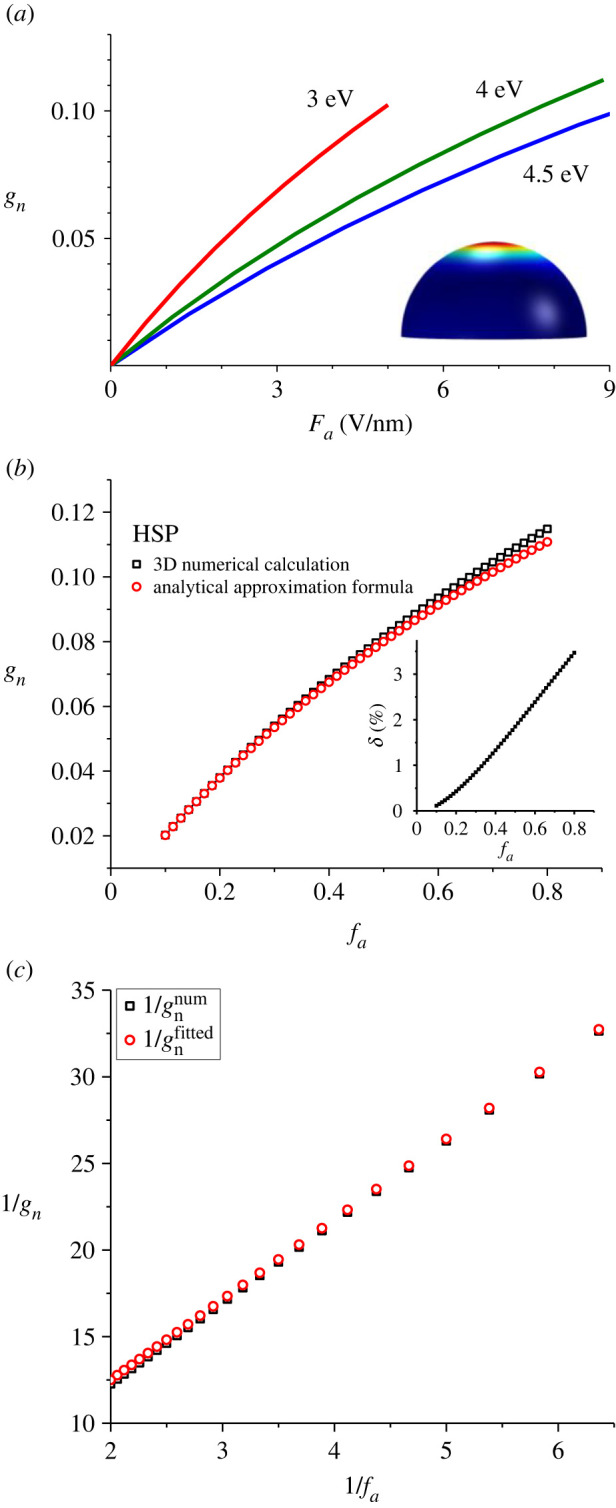
HSP emitter model. (*a*) Dependence of notional cap-area efficiency *g*_n_ on the apex value of local field magnitude, for three different values shown of local work function (inset—three-dimensional finite-element simulation of the electrostatic field for HSP). (*b*) The comparison (for a *ϕ* *=* 4.50 eV emitter) of numerically calculated values of notional cap-area efficiency and values of Jensen's analytical formula (inset—relative percentage difference *δ*). (*c*) To show (for a *ϕ* *=* 4.50 eV emitter) that 1/*g*_n_ is a nearly linear function of 1/*f*_a_: the black squares are the finite-element numerical results and the red circles are the corresponding values derived from equation (3.16).

A cylindrical simulation cell was used. The cell radius and height, the meshing geometry, and the electrostatic boundary conditions were chosen in accordance with the ‘minimum domain dimensions', meshing criteria and electrostatic arguments outlined in [37]. As an illustration, figure 1*a* shows the resulting field distribution for a particular case of the HSP model.

Integration of current density over the emitter surface, to evaluate the emission current, was carried out directly in the software package using the planar emission approximation and the kernel current density for the SN barrier.

### 4.2. Consistency check with Jensen's analytical treatment

For an initial comparison between our present results for the HSP model and those of Jensen, we show (in figure 1*a*) our results in the form used by Jensen in Fig. 30.3 of [19], namely a direct plot of *g*_n_ versus the apex field magnitude *F*_a_. (But note that Jensen uses a quantity measured in eV nm^−1^ to represent the behaviour of electrostatic fields: his quantity ‘*F’* equals our ‘*eF*’.) This is done for the three work-function values Jensen used. Our plot and Jensen's plot are closely similar.

For *ϕ* = 4.50 eV, figure 1*b* shows a more detailed comparison. The *percentage relative difference δ* between the analytical and simulation results is

4.1 $$\delta \equiv 100\text{\%}\times |g_{n}^{\mathrm{simulation}}\text{–}g_{n}^{\mathrm{analytical}}|/g_{n}^{\mathrm{analytical}}.$$

The inset to figure 1*c* shows how *δ* varies with *f*_a_: *δ* decreases almost linearly as the apex field magnitude *F*_a_ (and hence the scaled-field *f*_a_) increase. Over the ‘Pass’ range of the Orthodoxy Test (0.15 ≤ *f*_a_ ≤ 0.45), the percentage relative difference lies approximately in the range (–3% ≤ *δ* *≤* *–*1%).

Obviously, there is a question of whether this small difference is due to ‘approximation error’ in the analytical calculations or to ‘simulation error’. However, we know from the work of de Assis & Dall'Agnol [37], on minimum simulation domain dimensions, that the simulations ought to be highly precise. Thus, our expectation is that most of the difference *δ* arises from the mathematical approximations made in the analytical treatment.

For *ϕ* = 4.50 eV, figure 1*c* confirms the 1/*g*_n_ is a good linear function of 1/*f*_a_.

**Figure 2.**
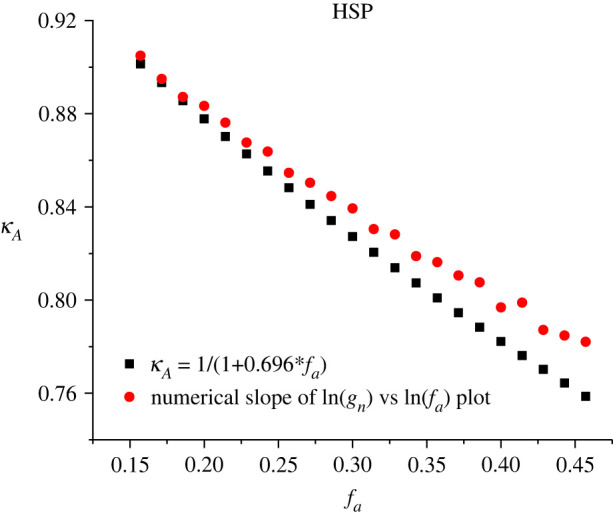
For the HSP emitter model, using the kernel current density for the SN barrier for simulations, with *ϕ* = 4.50 eV, the contribution *κ_A_* (to the total value of *κ*) resulting from field dependence in the calculated notional emission area. The figure compares the results of our finite-element electrostatic simulations (red circles) with the predictions of equation (3.19) (black squares).

A further comparison can be made between the plots of *κ_A_*(*f*_a_) derived from equation (3.16) and from our finite-element calculations. This is shown in figure 2. The degree of agreement is very satisfactory. More generally, figure 2 clearly predicts that *κ_A_* will be a noticeable function of *f*_a_ (and hence of apex field and of measured voltage). For the circumstances of these calculations, in the range 0.15 ≤ *f*_a_ ≤ 0.45, *κ_A_* takes values (for the SN barrier) in the approximate range 0.9 ≥ *κ_A_* ≥ 0.75.

## 5. Simulation results and discussion

The HSP model is the easiest to use when making comparisons between analytical models and simulations, and provides a useful degree of verification for the simulations. However, the HSP model is obviously not a realistic field emitter model. Other field emitter shapes, closer to experimental reality, are of greater interest. As indicated earlier, we have investigated three other emitter models: the HCP model, the HEP model and the spherically rounded (truncated) cone (SRC) model. For completeness, the data for the HSP model discussed in §4 are also included in diagrams below.

The current–voltage characteristics were calculated using the general procedure described in §4.1. As before, an emitter height of 4 µm and apex radius of 50 nm (corresponding to an apex sharpness ratio of 80) were used, and calculations were carried out for apex scaled-fields in the range 0.15 ≤ *f*_a_ ≤ 0.45.

**Figure 3.**
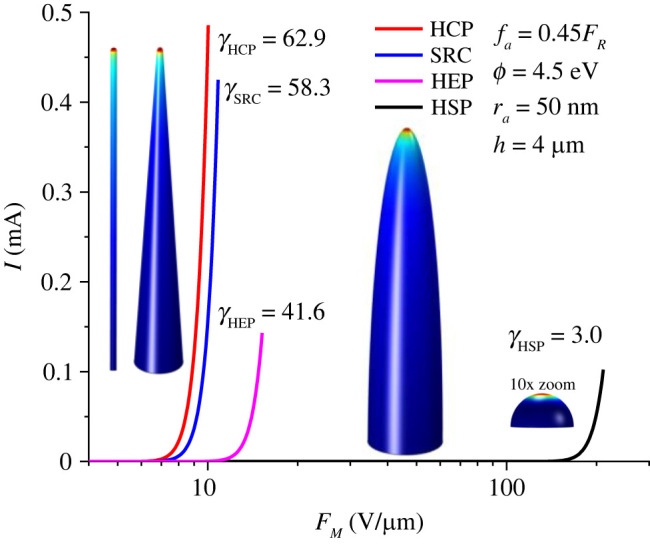
Data for the HSP, HEP, SRC and HCP models, for *ϕ* = 4.50 eV and an apex sharpness ratio (for the latter three models) of 80. The figure shows shapes, apex values of the field enhancement factor (*γ*), illustrative surface field distributions and plots of total emission currents as functions of the macroscopic field *F*_M_ applied between the top and bottom surfaces of the simulation box, over macroscopic field ranges corresponding to apex scaled-field values up to *f*_a_ = 0.45.

Figure 3 shows the results of the current density integration over the emitter surface, but with the current plotted as a function of the macroscopic field *F*_M_ that is applied between the top and bottom surfaces of the simulation box. The corresponding apex field enhancement factors (*γ*_a_) are also indicated.

As illustrated by the graphs, the lowest threshold values of macroscopic field (and hence of applied voltage) correspond to sharply pointed elongated shapes, as is well known. The shapes of individual carbon nanotubes correspond most closely to the HCP model, which is the model most effective at field enhancement. This is part of the reason why carbon nanotubes have been extensively investigated as field emitters in recent years.

**Figure 4.**
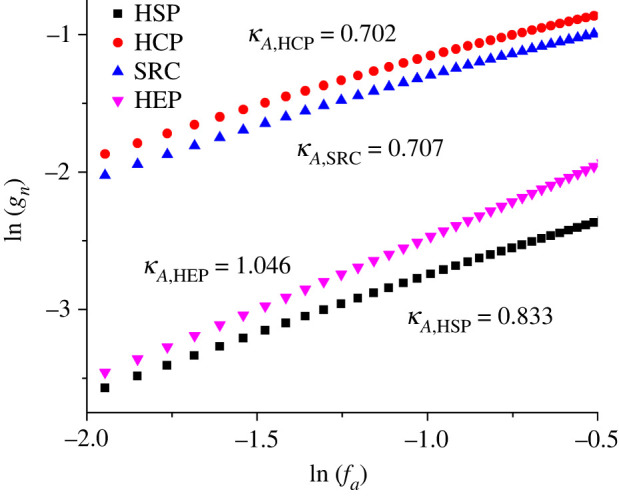
Dependences of the notional cap-area efficiency *g*_n_ on the (dimensionless) apex scaled-field *f*_a_, in the range 0.15 ≤ *f*_a_ ≤ 0.45, for the four model emitters investigated, as specified in figure 3. Estimates of effective values of *κ_A_*, derived from the mean slopes of these functions over the stated range, are shown.

Figure 4 shows logarithmically how the notional cap-area efficiency *g*_n_ varies as a function of apex scaled-field *f*_a_, for the four emitter shapes investigated. Clearly, these results fall into two groups. It might be expected intuitively that emitters with similar apex shapes would exhibit similar behaviour. However, the highest *g*_n_ values were in fact obtained for the HCP model.

This result was not obviously expected. However, closer examination shows that the HCP emitter not only has the largest apex FEF, but also has higher field values along a line along the surface, in a plane containing the model axis. This leads to greater integral current values and therefore to larger values of notional emission area (and hence to larger values of *g*_n_).

**Figure 5.**
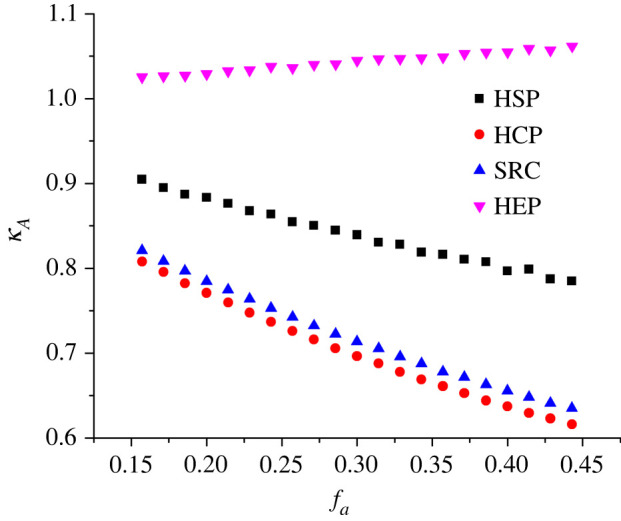
The contribution *κ_A_* (to the total *κ*-value) resulting from field dependence in the calculated notional emission area, for each of the emitter models investigated, as a function of the (dimensionless) apex scaled-field *f*_a_.

The quantity *κ_A_* represents the contribution (to the total *κ*-value) that is due to emitter-shape effects. To investigate numerically how *κ_A_* varies with *f*_a_ (and hence for electronically ideal emitters, with measured voltage), plots of dln{*g*_n_}/dln{*f*_a_} versus *f*_a_ were made for the studied tip shapes. These plots are shown in figure 5.

Figure 5 suggests that, for all tip shapes, there will be a contribution *κ_A_* (to the total *κ*-value) that is a function of the apex scaled-field *f*_a_ and hence of the independent variable in an AHFP-type equation. For the range 0.15 ≤ *f*_a_ ≤ 0.45 under discussion, table 2 records the variation in *κ_A_* and also its average value (*κ_A_*)_av_ over the range (obtained by linear regression to the data plots in figure 5).

**Table 2.** For the apex scaled-field range 0.15 ≤ *f*_a_ ≤ 0.45 and the four emitter shapes investigated (assuming an SN barrier), the limits of the variation of the contribution *κ_A_*(*f*_a_) across the range and its average value (*κ_A_*)_av_. Combining the individual four ranges provides an estimate of the total likely range of variation.

shape	*κ_A_*(*f*_a_)		(*κ_A_*)_av_
	*f*_a_ = 0.15	*f*_a_ = 0.45	
HSP	0.90	0.78	0.833
HEP	1.02	1.06	1.046
HCP	0.81	0.62	0.702
SRC(5°)	0.82	0.63	0.707
total range	1.02	0.62	n/a

## 6. A methodology for using *κ*-values in theory–experiment comparisons

With the theory and simulations described, we now present a new methodology for comparing FE theory and experiment. The methodology seeks to use an experimental *κ-*value to decide (if possible) which of two specific FE theories is ‘better science’. As an example, we shall seek to establish *experimentally* that the SN barrier used by Murphy and Good in 1956 is ‘better science’ than the ET barrier used by Fowler and Nordheim in 1928. Theoretically, there is no doubt about this (see [7]), but establishing a *decisive* proof from experiment has proved elusive (e.g. [38]).

We emphasize that the remarks here aim to establish a methodology and provide a ‘proof of concept’, but do not aim to produce a definitive result at this stage. We anticipate that getting a decisive result may be a long and messy process, but we hope that establishing this methodology may stimulate the development of techniques able to provide good experimental values of *κ*, with reliable error limits.

For the 1928 FN FE equation (as corrected in 1929) and the 1956 MG FE equation, both as expressed in AHFP mathematical form, table 3 collates the various individual contributions (as defined in §3.1) to the total AHFP exponent *κ*. Entries on line F could arise if an experimental emitter of interest were not adequately modelled by any of the emitter shapes we have examined (or not adequately modelled by the assumption of constant local work function), but at present we have no good knowledge of effects of this kind.

**Table 3.** Range limits on contributions to the total value of the AHFP equation pre-exponential parameter *κ*, and hence on its total value, for current–voltage characteristics taken from an electronically ideal, needle-shaped or post-shaped emitter, according to (a) 1928/1929 FN FE theory and (b) 1956 MG FE theory, for an emitter with uniform local work function 4.50 eV. (Contribution sources are defined in §3.1.)

contributions: sources of Δκ:	(a) FN (ET barrier)		(b) MG (SN barrier)	
	lower limit	upper limit	lower limit	upper limit
A:	2	2	2	2
B:	0	0	–0.77	–0.77
C:	–1	0	–1	0
D:	0.58	1.03	0.62	1.02
E:	∼0	∼0	∼0	∼0
F:	undefined	undefined	undefined	undefined
range of total *κ*:	1.58	3.03	0.85	2.25

**Figure 6.**
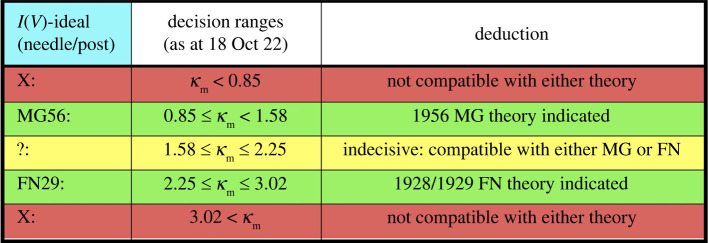
Decision table for interpreting an experimental value *κ*_m_ of the AHFP equation pre-exponential parameter *κ*, for current–voltage characteristics taken from an electronically ideal needle-shaped emitter or from a post-shaped emitter with apex sharpness ratio (height divided by apex-radius) 80, assuming a uniform local work function of 4.50 eV.

In each case, on the last line of table 3, we arrive at an estimate of the range within which *κ* is predicted to lie if the equation in question is an adequate representation of experimental reality. These two ranges can then be used to generate figure 6, which shows how an experimental estimate *κ*_m_ of the AHFP equation pre-exponential parameter *κ* is to be interpreted, for emitters of the kind under discussion. It is stressed that the numbers in this table are ‘first estimates’, based on our current theoretical assumptions, and may change as theoretical knowledge improves.

It is of interest to apply this decision table to the small number of relevant experimental *κ*_m_ values listed in table 1.

The Lauritsen value of 0 (on row 2) was a highly important early empirical result but has limited accuracy: it is also of note that the emission was coming from a small area (probably a pointed protrusion or low-work-function patch) on a cylindrical wire [28].

An objective of Stern *et al*.'s 1929 paper [8] was to determine whether the Lauritsen empirical value *κ* = 0 or the FN theoretical value *κ* = 2 better represented experimental reality. They concluded that the most appropriate existing dataset (taken over the largest voltage range) was that in Fig. 3 of the Millikan & Eyring's 1926 paper [29]. This dataset was also taken with a cylindrical wire emitter. Stern *et al*. concluded that taking *κ* = 2 gave the better fit to the experimental data. This conclusion was also supported (albeit less convincingly) by careful re-examination of some results of Gossling *et al.* [30] taken from a tungsten needle-like emitter (see Fig. 1 in [8] and related discussion). This Stern *et al*. conclusion led to the whole apparatus of FN plots, still in current use 90 years later.

However, if we apply the thinking of figure 6 above to the Stern *et al*. conclusion, then their result of *κ* = 2 is in the ‘*indecisive’* decision regime, thus apparently showing that the experimental data considered by Stern *et al*. are also compatible with 1956 MG FE theory. This amounts to a basic *experimentally derived* prediction that FN plots are not necessarily expected to be exactly straight, which is in accordance with modern theoretical thinking. (Exact straightness of an FN plot is predicted by 1928/1929 FN FE theory but not by 1956 MG FE theory.)

In 1939, the ‘precise measurements' by Abbott & Henderson reported in Part II of their paper led them to conclude that the best integral value was *κ*_m_ = 4 (see row 6). According to the decision table in figure 6, this result is not compatible with either FN FE theory or MG FE theory. This finding has never been satisfactorily explained and has usually been ignored. However, Abbott & Henderson were the first to point out (more than 80 years ago) that the value of *κ*_m_ must depend on the ‘exact microscopic shape of the point (emitter)’––as now well demonstrated by our present calculations.

Basic problems with all this early work are the limited accuracy of the experiments and the lack of realization that *κ*_m_ could legitimately be non-integral (e.g. [13]).

Turning to discuss modern work on *κ*_m_, the only table entry (on row 11) is to the Ioffe Institute work on LAFEs built from carbon nanotubes. The use of LAFE data is not ideal, but these are the only experimental datasets that we currently have access to that have been controlled and recorded electronically (which is necessary for precise extraction of *κ*_m_-values). If we make the usual assumption that traditional metal emission theory is ‘good enough’ for CNT data analysis, then applying figure 6 thinking to the row 11 result that *κ*_m_ = 1.65 yields the conclusion that these experiments are marginally indecisive.

In summary, the existing experimental evidence, as listed in table 1, does not enable us to reach a decisive experimentally based decision that 1956 MG FE theory is a better description of experimental reality than 1928/1929 FN FE theory, notwithstanding that it is theoretically certain that the former is ‘better physics’ than the latter. Thus, there is a strong need for appropriate and accurate new experiments, preferably on metal emitters.

## 7. Summary and discussion

The main focus of this research has been the prediction of values for the pre-exponential parameter *κ* in the AHFP mathematical equation, and on preliminary comparison of predicted values with such experimental results as currently exist. So-called EMG FE theory has been outlined, six main theoretical contributions to *κ* (for current–voltage characteristics taken from electronically ideal FE systems) have been identified and existing reliable knowledge about these contributions (as far as we are aware of it) has been collated into table 1. A gap in this knowledge, namely the detailed effect of emitter shape on the related contribution *κ_A_*_,_ has been filled (at least partially) by simulations on four different emitter tip shapes, for the specific common work-function value 4.50 eV. These shapes include the HSP model, for which there is an analytical treatment by Jensen. For this model, our simulations and Jensen's analytics are in very good agreement.

A new methodology for using an experimental value *κ*_m_ of the pre-exponential exponent *κ* in the AHFP mathematical equation to decide which of two specific theories of electronically ideal FE current–voltage characteristics is ‘better science’ has been outlined and applied to the choice between the 1928/1929 FN and 1956 MG FE theories. Based on current theoretical knowledge, the relevant decision table (figure 6) has been constructed and applied both to historical values of *κ*_m_ and to the one modern value that we consider worth discussing.

As indicated above, an interesting result relates to the Stern *et al*. analysis of one dataset reported by Millikan & Eyring and two datasets reported by Gossing (all taken in 1926). Stern *et al*. concluded that taking *κ*_m_ = 2 gave lower residual error than taking *κ*_m_ = 0, and hence that the results were compatible with 1928/1929 FN FE theory. As already indicated, the whole apparatus of FN plots, still in use 90 years later, was built on this finding. But the decision table in the present paper shows that the 1929 conclusion that *κ*_m_ = 2 is also compatible with 1956 MG FE theory and hence indicates *from experimental analysis* that FN plots are not the only suitable option for analysing FE current–voltage data. (An alternative is the so-called MG plot [11], introduced on the basis of theoretical arguments.)

The single modern estimate of *κ*_m_ in the table tends to favour 1956 MG FE theory rather than 1928/1929 FN FE theory, but is marginally indecisive. Thus, FE science still remains in the state in which it has been since the late 1950s: it is theoretically certain that MG FE theory is ‘better physics’ than FN FE theory, but demonstrating this experimentally by a decisive argument remains elusive. The most important need looks to be for better experimental methodologies for accurate measurement of *κ*_m_ for metal emitters and for determining reliable error limits.

We make again the general point that this paper represents ‘first steps’ toward a new methodology. As theoretical knowledge improves, and experimental results get better defined and more accurate, the decision table will change numerically (and perhaps new contributions to *κ* will be added), and it is expected that its interpretation will change qualitatively. When we have reached the point that this methodology can clearly show, in alignment with theoretical understanding, that 1956 MG FE theory is ‘better science’ than 1928/1929 FN FE theory, then it will be timely to construct one or two new decision tables that compare 1956 MG FE theory with one or two more sophisticated FE theories—for example, the 2014 Kyritsakis–Xanthakis theory [39] of emission from an earthed sphere.

We note that our simulations have so far been carried out only for one value of work function and one value of apex sharpness ratio. This has been sufficient to establish a proof of concept for our new methodology, but we also plan to carry out further simulations for ranges of values of these parameters.

Finally, two recent papers by other authors need comment. For a model involving a hemi-ellipsoid-on-cylindrical-post (HECP model), Ramachandran & Biswas [26] have investigated how the dependence of the notional emission area on the apex field varies with the ratio ‘hemi-ellipsoid height divided by apex-radius’. Detailed investigation of the relationship between their results and ours is outside the scope of this paper, but our intention is to incorporate an appropriately customized version of their results into updated versions of tables 2–4, in due course.

In the second recent paper, Ayari *et al*. [40] discussed whether methodology based on measuring *κ*_m_ could be used to extract a value for the local work function *ϕ* from FE current–voltage measurements. We agree with their conclusion that this would be a very challenging task, but we think that the discussion in the present paper shows that the complete theoretical picture is more complex than their arguments have assumed (although the conclusion would be the same).

Finally, we state again our belief that the decision table methodology introduced here could become a useful tool in developing better FE science.

## Data Availability

Computations described in this paper have been carried out using two standard commercial Software packages. COMSOL has been used to carry out electrostatic modelling. LABVIEW has been used for calculation of emission current densities and emission currents, and related analyses. When COMSOL is used, a large internal data file is generated by COMSOL and passed to LABVIEW. Appropriate modelling information (as described in the main text) and appropriate sequences of input instructions have to be entered into the software packages, but there are no large data input files and there has been no need for low-level code to be written. It is assumed that researchers able to drive the software packages used (or packages with equivalent functionality) are able to generate the package input instructions needed to implement the procedures described in the main text or in the electronic supplementary material, and to generate associated diagrams. Additional details concerning the electrostatic modelling, beyond those provided in the main text, are essentially similar to those provided as electronic supplementary material to [11], which can be found via the link: https://doi.org/10.6084/m9.figshare.c.5324958. The main difference is that in the present paper four different emitter shapes were analysed, rather than the single shape treated in [11].
